# Supplementary material for: MicroRNA750-3p Targets Processing of Precursor 7 to Suppress Rice Black-Streaked Dwarf Virus Propagation in Vector Laodelphax striatellus
Source: Viruses. 2024 Jan 8;16(1):97. doi: 10.3390/v16010097 (PMC10820416; doi:10.3390/v16010097)
Supplement: Supplementary file 1 [file viruses-16-00097-s001.zip › viruses-2764945-supplementary.pdf]

Table S1. Primers used in this research

Primer Name	Sequence
miR750-3p-RT	GTCGTATCCAGTGCAGGGTCCGAGGTATTCGCACTGGATACGACTTTAGCT
miR750-3p-F	GCGCCCAGATCTAACTCTTCCA
miR-R	CAGTGCAGGGTCCGAGGTAT
RBSDV P10-qF	GCCCCACGTTGCATCTTC
RBSDV P10-qR	TGTTGGGCAAAGTGCTAGTTTC
CDPK-qF	TTGGAACTGGTGCCTTTTCG
CDPK-qR	GTCCGCTTGGTAGACTCACT
Neur-qF	AGACCCATGAAGACCACTCG
Neur-qR	ATACAACCTCGCCCGAAC
Trans qF	GTCGTCAACTGGAGGCTACT
Trans qR	GATGTTCTGCTCGACGTGAC
HSP60 qF	GGCGTTGACATTCTTGCTGA
HSP60 qR	ATCTTGAACCAATCTCGCGC
POP-qF	CCTAACTGCTTTCATCGGCC
POP-qR	GAGTCGGCTGCTTGGTTATG
dsPOP-F	TAATACGACTCACTATAGG GATCCCATTTTGGATATT
dsPOP-R	TAATACGACTCACTATAGG AGGCCCAAATCGCAGTT
dsGFP-F	TAATACGACTCACTATAGGCGACTTCTTCAAGTCCGCCA
dsGFP-R	TAATACGACTCACTATAGGCTCAGGTAGTGGTTGTCGGG
